# Does adjuvant chemotherapy change bone mineral density and related serum biomarkers in women with breast cancer? 

**DOI:** 10.22088/cjim.8.2.91

**Published:** 2017

**Authors:** Reza Safaei-Nodehi, Javad Esmaili, Ramazanali Sharifian, Shafieh Movaseghi, Sayeh Parkhideh

**Affiliations:** 1 Hematology- Oncology and BMT center, Tehran University of Medical Sciences, Tehran, Iran.; 2 Department of Nuclear Medicine, Imam Khomeini Hospital, Tehran University of Medical Sciences, Tehran, Iran.; 3 Rheumatology Research Center, Tehran University of Medical Sciences, Tehran, Iran.; 4 Taleghani Bone Marrow Transplantation Center, Taleghani Hospital, Shahid Beheshti University of Medical Sciences, Tehran, Iran.

**Keywords:** Bone mineral density, Breast cancer, Chemotherapy, Z-score

## Abstract

**Background:**

The primary objective of this study was to assess BMD change in Iranian females with breast cancer.

**Methods:**

A sample of 73 female breast cancer patients treated with adjuvant chemotherapy either alone or followed by radiotherapy between March 2013 and February 2016 were considered for this study. Bone mineral density (BMD) change was evaluated by measuring z-score of lumbar spine, femoral neck (right and left side) as well as biochemical measurements. With respect to WHO categorization for the treatment of osteoporosis, patients were categorized as normal BMD, osteopenic and all analysis was done separately.

**Results:**

In women with normal BMD, lumbar spine and femoral neck (right side) z-score decreased significantly by 15.7 and 39%, respectively (p<0.05). In osteoporosis group, there was no BMD change in any of the lumbar or femoral neck z-scores. However, in osteopenic patients, femoral neck BMD decreased significantly by 40.9% after 8 months (p=0.003). The level of all measured biomedical markers such as Ca, Alb, P and vitamin OHD decreased significantly in a follow-up visit in both osteoporosis and normal BMD.

**Conclusion:**

Our results revealed that adjuvant chemotherapy led to unfavorable effects on lumbar spine and femoral neck means z-score during 8 months. Also, unfavorable changes in biochemical markers appeared in all groups.

Epidemiologic studies show that breast cancer (BC) is the second most common cancer among females. The incidence rates of BC vary from 27 per 100,000 person in East Asia and Middle Africa to 96 per 100,000 person in Western Europe (1). Women with early stage BC were often not given clinical guideline recommended adjuvant chemotherapy (2-4). Unfortunately, chemotherapy that reduces estrogen levels can affect bone mineral density (BMD) strengths by increasing bone resorption without an equivalent increase in bone formation that could increase the risk of osteoporosis (5-7). Similarly, several studies have shown the positive association between BMD and breast cancer (5, 6, 8, 9). In one of these studies in premenopausal women with BC during 6 months of chemotherapy, the reduction of both lumbar spine and hip BMD was approximately 4 and 2%, respectively (10). It has been estimated that postmenopausal women with breast cancer lose 2-3 fold more BMD compared to healthy postmenopausal women (7). In addition, some other studies reported the independent relation between BC and BMD (11-13). Among the different side effects of chemotherapy (14), the reduction of blood biomarkers play an important role in BMD.

A longitudinal study in adults has shown that calcium intake as a predictor, has a significant effect on total bone mass. Results from the National Health and Nutrition Examination Survey (NHANES) show that Caucasian women who drink low milk during childhood and adolescence period, had low fat BMD during adulthood and a higher risk of fracture (15). Also, the effect of adequate vitamin D intake is essential for skeletal health. Nevertheless, the predictive value of vitamin D levels in patients with breast cancer is not clear (16). Since the quality of life and survival in patients with BC is improved with adjuvant chemotherapy and with respect to the different findings, we investigated a prospective study to address the effect of chemotherapy on bone mineral density as well as blood biomarker changes in pre- and postmenopausal women with breast cancer.

## Methods

**Study population:** This prospective, single-center study was done in the departments of Oncology, Hematology and Medical Oncology, Cancer Research Institute and Nuclear Medicine Department, Imam Khomeini Hospital Complex, Tehran, Iran. All breast cancer patients (stages I, II, and operable IIIA) over 18 years (either pre or post- menopausal period) who were treated with adjuvant chemotherapy either alone or followed by radiotherapy were entered into this study. Patients with a history of bone disease, previous chemotherapy or radiotherapy of the lumbar spine or femoral neck, corticosteroid treatment or any other conditions which could affect bone density negatively, were excluded from the study. A sample of 73 women with BC who met the inclusion criteria between March 2013 and February 2016 entered the current study. 

Each patient was prospectively evaluated on two occasions: at baseline (the initiation of chemotherapy, which was about 2-4 weeks after breast surgery) and at 8 months after their last round of chemotherapy (follow-up visit). With respect to World Health Organization and study protocol, patients with t-score between −1.0 and −2.5 were categorized as osteopenia and received a fixed dose of calcium- D (1000-1200 mg/day). 

**Figure 1:**
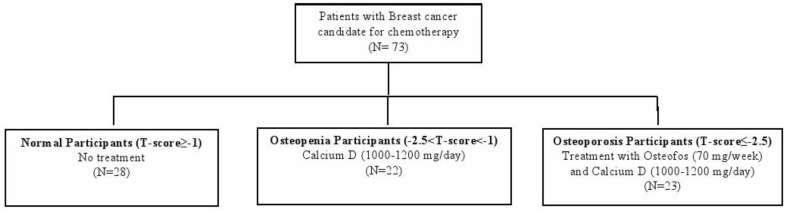
Allocation scheme of the patients

Patients with osteoporosis (t-score ≤ -2.5) received both Osteofos (70mg/week) and calcium- D (1000-1200 mg/day). Normal BMD was defined as t-score ≥-1.0 (17). Therefore, the patients were categorized into three groups; osteopenia (n=23), osteoporosis (n=22) and the normal BMD group (n=28) (figure 1). 

The Ethics Committee of the Research Institute of Cancer Sciences and Nuclear Medicine Department, Tehran University of Medical Sciences approved the design of the study, and all participants provided written informed consent.

**BMD measurement:** Bone mineral density (g/cm2) at the lumbar spine (L1-L4) and femoral neck (right and left side) was measured by dual energy x-ray absorptiometry (DEXA) using a prodigy densitometer (GE-Lunar, Milwaukee, USA). BMD was assessed as z-score (the standard deviation (SD) from the mean for age-matched weight adjusted for body mass) or t-score (SD from the mean for young women). Machine calibration and quality assessment-tests were done daily and monthly. BMD measurement at baseline and follow-up visit were carried out by the same machine. 

**Medical history and laboratory measurements: **The order of postoperative chemotherapy was made by the patient’s oncologist with respect to the size and features of the tumor. BMD measurements at baseline and follow-up visit were done and reported by a nuclear medicine specialist. Information about menstrual or menopausal periods was determined based on a self-reported questionnaire at the time of bone density measurement. All laboratory measurements were venous samples drawn after 12-14 h overnight fasting between 7:00 and 9:00 A.M into vacationer tubes that centrifuged within 30-45 minutes after collection. Both inter were- and intra-assay coefficients of variation (CV) at baseline and follow-up visit were less than 2.3%. 

Calcium (Ca) was assayed by complex colorimetery using o-cresolphthalein using Pars Azmon kits (Pars Azmon Inc., Tehran, Iran). Alkaline phosphate was measured by enzymatic colorimetery by xylidyl blue (Pars Azmon kits).Vitamin OHD levels were measured by patients’ serum 25-hydroxyvitamin D3 (vitamin OHD) levels using enzyme immunoassay (EIA) kits from DRG Co (USA). Albumin (Alb) was assayed by serum protein electrophoresis method. The whole samples were analyzed when internal quality control reached the acceptable criteria.

**Statistical analysis: **To better understand the mechanism of the effects of chemotherapy on BMD change, the analysis was done separately for osteopenia, osteoporosis and normal BMD groups. Mean (SD) for normal distributed values was continuous and frequencies (%) for categorical variables were described at baseline and during a follow-up visit. For skewed variable median, interquartile (IQ range) is expressed. Measurement within groups changes in the parameters with normal distribution which was performed using paired sample t-test while Wilcoxon signed-rank tests for skewed variables. Difference for categorical variables was evaluated using chi-square test. 

Between group changes were tested with one-way ANOVA or Kruskal-Wallis as appropriate. All statistical analysis was performed using SPSS Version 20, with a two tailed p<0.05 considered significant.

## Results

Seventy-three breast cancer patients with the mean of 45.0(11.2) years were considered for this study. Totally, 22 (31.5%) patients had osteopenia, 23 (30.1%) patients had osteoporosis and 28 (38.4%) patients had normal BMD. Women in osteoporosis group were older, 50 (40-59) than the osteopenia or normal BMD groups, but this difference was not statistically significant (P=0.07). We discovered 24 (32.9%) females experienced menopause. Of these, 56.5% were osteoporotic followed by osteoponic and individuals with normal BMD (P=0.01) (table 1).

**Table 1:** Comparison of baseline characteristics between patients with normal BMD and osteopenia

	**Age, years**	**Menopause, yes**
Total population (n=73)	43.0 (36.5-56.0)	24 (32.9)
Normal BMD (n=28)	41.5 (38-47)	5 (17.9)
Osteopenia (n=22)	39.5 (30-50)	6 (27.3)
Osteoporosis (n=23)	50.0 (40-59)	13 (56.5)
P-value	0.07	0.01

Age was shown as median (interquartile range) and p-value according to Kruskal Wallis test; n% was shown for categorical variables with p-value according to chi-square.

Comparison results of Z-score of BMD at baseline and follow-up visit have been illustrated in table 2. The mean of the total lumbar spine (-1.02 vs. -1.21, P=0.003) and femoral neck (right side) (-0.25 vs. -0.41, P=0.02) decreased after 8 months in a normal BMD group. 

Left femoral neck (BMD) was higher in follow-up visit but this change was not significant. Among patients with osteoporosis, there were no significant changes in lumbar and femoral neck (right and left) between baseline and follow-up visit. Right femoral neck (BMD) decreased significantly (0.13 to -0.22, P=0.003). 

**Table 2:** Comparison of BMD z-score in the lumbar spine, femur neck at baseline and after 8 months follow-up with respect to BMD groups

	**Lumbar spine**	**Femoral neck** **(Left side)**	**Femoral neck** **(Right side)**
**Normal BMD**			
Baseline visit	-1.02(0.85)	-0.29(0.85)	-0.25(0.81)
Follow-up visit	-1.21(0.9)	-0.19(0.43)	-0.41(0.89)
P-value	0.003	0.37	0.02
**Osteopenia**			
Baseline visit	-1.67(0.45)	-0.4(1.11)	0.13(0.93)
Follow-up visit	-1.73(0.41)	-0.56(1.13)	-0.22(0.95)
P-value	0.24	0.07	0.003
**Osteoporosis**			
Baseline visit	-2.82(1.06)	-1.53(1.36)	-1.05(1.23)
Follow-up visit	-2.78(0.88)	-1.64(1.29)	-1.39(1.34)
p-value	0.59	0.25	0.09

Mean (SD) was shown for continuous variables and p-value was calculated using paired sample t-test or Wilcoxon signed rank test.

Nonetheless, this change was not significant for left femoral neck or total lumbar. The level of all measured biomedical markers such as Ca, Alb, P and vitamin OHD decreased significantly in follow-up visit (after 8 months chemotherapy) in both osteoporosis and normal BMD. The reduction level of albumin and vitamin OHD was not significant in osteoporosis patient group (table 3).

**Table 3:** Comparison of serum biomedical markers at baseline and after 8 months follow-up with respect to BMD groups

	**Calcium** **(mg/dL)**	**Phosphate** **(mg/dL)**	**Albumin** **(mg/dL)**	**Vitamin OHD(ng/dl)**
**Normal BMD**				
Baseline visit	8.69(0.45)	4.05(0.66)	4.52(0.82)	12.07(4.7)
Follow-up visit	8.53(0.37)	3.76(0.47)	4.16(0.67)	10.71(3.94)
p-value	0.035	<0.001	<0.001	0.05
**Osteopenia**				
Baseline visit	8.82(0.43)	3.96(0.55)	4.58(0.7)	10.24(3.49)
Follow-up visit	8.59(0.33)	3.79(0.56)	4.4(0.59)	10.0(3.71)
P-value	0.002	0.01	0.07	0.53
**Osteoporosis**				
Baseline visit	8.65(0.43)	3.89(0.52)	4.48(0.49)	11.96(4.63)
Follow-up visit	8.42(0.35)	3.64(0.55)	4.24(0.34)	10.43(4.34)
P-value	0.002	0.003	0.018	0.008

Mean (SD) were shown for continuous variables and p-value was calculated by paired sample t-test or Wilcoxon signed rank test

The proportion of the changing direction and mean change percentage were shown in table 4 with respect to different groups. A considerable negative change in BMD after 8 months follow-up was notable. The mean level of the right femoral neck in osteopenia group decreased by 40.9% while these changes were 39 and 24.5% in normal BMD and osteoporosis groups, respectively. Our results show that the mean change level of the left femoral neck in normal BMD group increased by 52.6%, but this was not statistically significant. 

**Figure 2:**
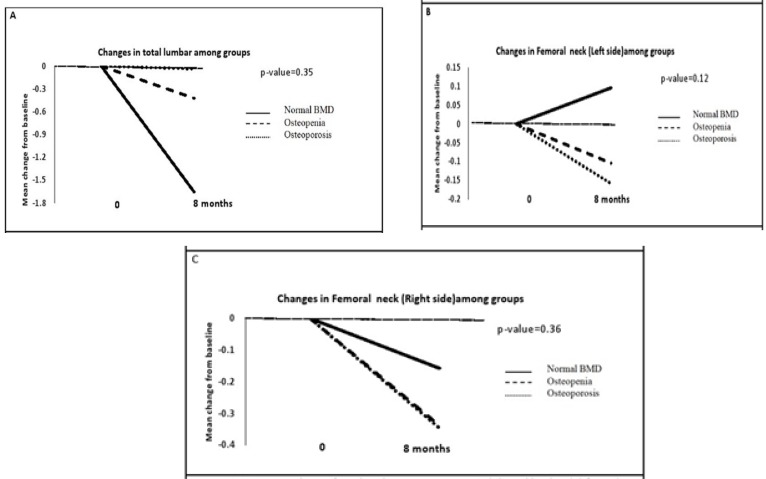
BMD mean change from baseline among groups in (A) total lumbar (B) femoral neck (left side) (c) femoral neck (right side

**Figure 3:**
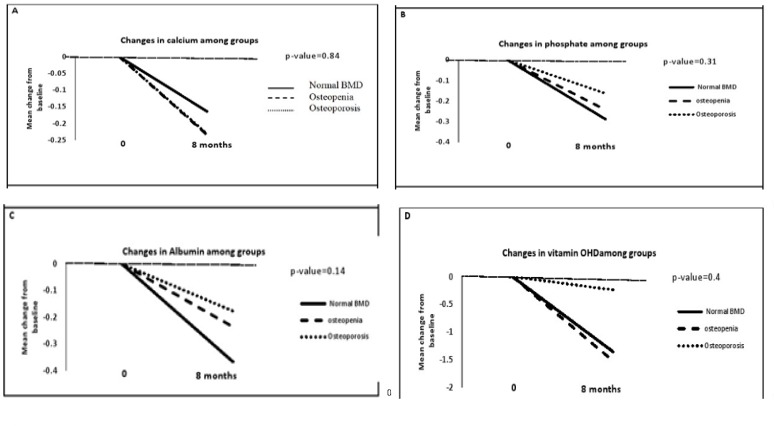
Serum biochemical markers mean change from baseline among groups in (A) calcium (B) phosphate (c) Albumin (D) vitamin OHD

We also found that the mean level of vitamin OHD decreased by 14.7% in osteoporosis group. The mean changes of total lumbar, right and left femoral neck from baseline for different groups were shown in figure 2 (A-C). Comparisons of these changes show that there is no significant difference among these groups. Different patterns for mean changes of biochemical markers among the three groups were shown in figure 3 (A-D). These changes were not statistically significant.

**Table 4:** The proportion of the changing direction with respect to BMD groups

	**No change** **n (%)**	**Negative change** **n (%)**	**Positive change** **n (%)**	**Mean Change ** **(%)**
**Normal BMD**				
Spin BMD(gr/cm2)	1(3.6)	7(25.0)	20(71.4)	-15.7
Femur BMD(gr/cm2) (Left side)	1(3.6)	15(53.6)	11(40.7)	+52.6
Femur BMD(gr/cm2) (Right side)	1(3.6)	7(25.0)	18(64.3)	-39.0
Calcium(mg/dL)	0	7(25.0)	21(75.0)	-1.9
Phosphate(mg/dL)	0	3(10.7)	25(89.3)	-7.7
Albumin(mg/dL)	0	1(3.6)	27(96.4)	-8.6
Vitamin OHD(ng/ml)	0	8(28.6)	20(71.4)	-12.7
**Oteopenia**				
Spin BMD(gr/cm2)	1(4.5)	9(40.9)	12(54.5)	-3.5
Femur BMD(gr/cm2) (Left side)	0	8(36.4)	14(63.6)	-28.6
Femur BMD(gr/cm2) (Right side)	1(4.5)	3(13.6)	17(77.3)	-40.9
Calcium(mg/dL)	0	3(13.6)	19(86.4)	-2.7
Phosphate(mg/dL)	0	4(18.2)	18(81.8)	-4.5
Albumin(mg/dL)	0	6(27.3)	16(72.7)	-4.1
Vitamin OHD(ng/ml)	1(4.5)	8(36.4)	12(54.5)	-2.4
**Osteoporosis**				
Spin BMD(gr/cm2)	0	8(34.8)	15(65.2)	+1.4
Femur BMD(gr/cm2) (Left side)	2(8.7)	7(30.4)	14(60.9)	-6.7
Femur BMD(gr/cm2) (Right side)	0	8(34.8)	15(65.2)	-24.5
Calcium(mg/dL)	0	5(21.7)	18(78.3)	-2.7
Phosphate(mg/dL)	0	5(21.7)	18(78.3)	-6.7
Albumin(mg/dL)	0	6(26.1)	16(69.6)	-5.7
Vitamin OHD(ng/ml)	0	6(26.1)	17(73.9)	-14.7

## Discussion

To our knowledge, currently this is the first study in the Middle East which illustrates the mean change level of bone density for age (z-score) in patients with breast cancer after chemotherapy. Our findings revealed that adjuvant chemotherapy led to unfavorable effects at both lumbar spine and femoral neck, the mean z-score resulted in 15.7% and 39% decrease, respectively in normal BMD group at 8 months TR. This reduction was notable on the femoral neck of osteoporosis patients (40.9%). We also showed that blood biochemical markers in women, who were treated with adjuvant chemotherapy, decreased significantly from baseline. 

Numerous epidemiologic studies have demonstrated that BMD levels decrease after chemotherapy in patients with BC (5, 6, 8, 9). Greep et al. in a study of 130 women with BC showed that the mean adjusted bone density in both spine and hip was significantly (p<0.05) lower in patients who received chemotherapy. In spite of that, they mentioned that the causal effect of chemotherapy is not specified (6). Moreover, Cameron et al. showed that loss of both spine and hip BMD in pre-menopausal women with breast cancer after adjuvant chemotherapy was not dependent on ovarian function (10). Additionally, there is the evidence of an inverse association between bone mineral density and the risk of breast cancer in an Israeli population. Their study indicated higher z-score at the femoral neck and total hip (5). Our findings for patients with normal BMD at baseline provide additional support of a decreased z-score of both the lumbar and femoral neck (right side). 

Markopoulos et al. (18) assessed the effect of anastrozole on BMD change at the hip and lumbar spine in 213 women with positive history of breast cancer. Women were randomized to receive anastrozole alone or with risedronate based on their t-score. All patients received 1000 mg calcium and 400 IU vitamin D daily. After 12 and 24 months, BMD loss appeared only in the anastrozole-treated groups with a t-score ≥ -1 was 5.3% and 2.5% at the lumbar spine. Women receiving anastrozole with t-scores -2<T<-1, lost 0, 1.5% BMD at the lumbar spine.

 Mohamed et al. (19) in postmenopausal Egyptian women showed that biochemical markers(such as Ca, vitamin OHD and alkaline phosphatase) decrease bone formation and resorption after adjuvant chemotherapy. In this regard, most, but not all studies show the positive association between low vitamin D and risk of breast cancer (20-24). Additionally, Datta and Schwartz (7) in a study from 16 trails of Ca±vitamin OHD, showed that commonly recommended doses of Ca (500-1500 mg/day) and vitamin OHD (200-1000 IU/day) were not sufficient to prevent bone loss in pre- and postmenopausal women with breast cancer. In keeping up with other findings, our results show that the level of biomarkers especially Ca decreased approximately by 2.4 % in normal, osteopenia and osteoporosis groups. We also found that the level of vitamin OHD dropped in normal BMD and osteoporosis (12.7 and 14.7%, p<0.05) categories, but this reduction was not significant in osteopenic patients (2.4%, P=0.53). 

With respect to National Osteoporosis Foundation guidelines, consideration of pharmacological treatment for osteopenic patients is helpful and prevents further bone loss (25). In the current study, we showed that pharmacological treatment with calcium-D and Osteofos caused a non-significant decrease in BMD change after 8 months of chemotherapy in patients categorized as osteoporotic. With respect to results of patients with osteopenia, commonly prescribed doses of calcium-D is not sufficient and it seems further treatment is required to prevent BMD and biomarker reduction. Yet the controversial effect of supplemental Ca intake on the increasing risk of cardiovascular disease from BC treatment should be considered (7). 

More than 50 percent of the blood serum proteins comprised serum albumin. Reduction of albumin level is an independent predictive indicator of breast cancer (26). In the current study, a marked albumin level reduction was observed in both normal BMD and osteopenia during chemotherapy. The reduction in osteoporosis was not statistically significant in the level of 0.05. This could be due to small sample size of the assigned categories. The strength of the present prospective study is that, it is the first study of BMD change in different groups in patients with BC in the Middle Eastern region. Furthermore, these findings are clinically important and could be used by clinicians and pharmacologists. 

Some limitations of the current study are needed to be addressed. Anthropometric measurements such as BMI (27), family history of breast or other skeletal disorders were not measured. Despite that, our aim did not examine the effect of the known risk factors affecting BMD change. This is the reason pathway and mechanism of these changes that were not evaluated in the current study. Besides, BMD change on patients with breast cancer who did not receive chemotherapy is probable. As a consequence, further studies as well as clinical trials are needed to assess these associations in women with breast cancer.

In conclusion, the clinical evaluation of total lumbar and femoral neck z-score as well as biochemical markers shows that adjuvant chemotherapy was responsible for these changes in a decreasing manner in women with breast cancer. Since the level of vitamin D decreased after chemotherapy, hence, monitoring may be valuable for skeletal health. Overall, further studies are needed to evaluate the relationship between BMD and vitamin D in women with breast cancer as a clinical point of view.
